# Tobacco use patterns in traditional and shared parenting families: a gender perspective

**DOI:** 10.1186/1471-2458-10-239

**Published:** 2010-05-10

**Authors:** Joan L Bottorff, Mary T Kelly, John L Oliffe, Joy L Johnson, Lorraine Greaves, Anna Chan

**Affiliations:** 1 Faculty of Health and Social Development, University of British Columbia, Kelowna, British Columbia, Canada; 2 Nursing and Health Behaviour Research Unit, University of British Columbia, Vancouver, British Columbia, Canada; 3 School of Nursing, University of British Columbia, Vancouver, British Columbia, Canada; 4 System Strategy Division, Ministry of Health and Long-Term Care, Province of Ontario, Canada; 5 Nursing and Health Behaviour Research Unit, University of British Columbia, Vancouver, British Columbia, Canada

## Abstract

**Background:**

Although researchers have focused on women's smoking during pregnancy and the postpartum period and the influence of household interactions on their tobacco reduction efforts, little attention has been given to parents' efforts to regulate smoking during the child-rearing years. The objective of this study was to examine how parenting young children and gender relations reflected in couple dynamics influence household tobacco use patterns and, specifically, women's tobacco reduction efforts.

**Methods:**

As part of a longitudinal, grounded-theory study with 28 couples to examine the place of tobacco in the lives of new parents, each parent participated in one or two individual, semi-structured interviews during the first three years postpartum. Grounded theory methods and a gender relations framework were used to analyze transcribed data.

**Results:**

Two different parenting styles that couples adhered to were identified. These parenting styles reflected performances of femininities and masculinities, and were associated with particular smoking patterns. *Traditional parenting *reinforced by women's alignment with emphasized femininities and men's alignment with hegemonic masculinities placed women with smoking partners at risk for relapse. Women's actions to be supportive partners facilitated couples' continued smoking. In *shared parenting *dyads, egalitarian practices tended to support successful transitions to smoke-free homes. Women's ability to exert more influence around family decision making, and the acceptance of new masculine identities associated with fatherhood were influential. In non-smoking dyads where the mother, father, or both reduced or stopped smoking, we observed a subtext of potential conflict in the event either the mother or father relapsed.

**Conclusions:**

Decisions about tobacco use are made within relationships and social contexts that vary based on each individual's relationship to tobacco, divisions of domestic labour and childcare, and other activities that impact tobacco use. Sensitive approaches to tobacco reduction for women and men must be developed building on greater understanding of gender relations and how tobacco use is integrated in spousal and parental roles.

## Background

The ideals of contemporary parenting now carry the social expectation that smoking is incompatible with being a good parent, especially for mothers [1]. Despite this anti-smoking social climate, in Canada smoking prevalence among young adults aged 20-24 years has increased to 27% [2], exceeding the 21% prevalence rate among 24-35 year olds, and increasing the likelihood of smoking during child-bearing years and the health risks of tobacco exposure for young children. Although researchers have focused on women's smoking during pregnancy and the postpartum period and the influence of household interactions on their tobacco reduction efforts [3,4], less attention has been given to parents' efforts to regulate household smoking during early child-rearing years. Reviews of intervention studies to reduce parents' smoking prevalence or children's exposure to secondhand smoke have indicated that there is little conclusive evidence regarding effective interventions [5,6] and that changes in parental smoking are difficult. It has been suggested that effective approaches to support tobacco reduction among parents and smoke-free homes need to be based on an appreciation of the everyday realities of family life and the scope parents have to make changes [7]. The first three years following the birth of a child is a time of tremendous challenges for families that involves personal, care-giving, and work-related changes that influence gender relations. Parents' decisions about tobacco use are made in this evolving interpersonal and social context. A better understanding of how these dynamics influence tobacco use is necessary for developing gender sensitive interventions to support families in establishing smoke-free homes.

### Gender relations, masculinities, femininities and tobacco use

When conceptualizations of gender relations are applied to health the focus is on the interplay within and between genders, as bound by specific settings and contexts, and the way these relations influence health behaviours [8]. Theories of gender as a social construct and practice draw attention to the relational and intra-relational nature of masculinities and femininities. In Howson's [9] analysis, hegemonic masculinity dominates all other gender practices, and masculinities and femininities are constructed as relations of alliance or contestation. Howson conceptualizes femininities in tripartite terms, abstracting *emphasized*, *ambivalent *or *protest *femininities. *Emphasized *femininity is complicit with, and accommodates, hegemonic masculinity. *Ambivalent *femininities represent a strategic combination of resistance and cooperation with hegemonic masculinity. *Protest *femininities challenge the foundation of the gender order, questioning the assumptions of alliance and complicity that order the intra-relational constructs configuring masculinities and femininities. Similarly, a plurality of masculinities - *complicit, marginalized and subordinate *operate in relation to hegemonic masculinity [9]. *Complicit *masculinities sustain hegemony by trading on traditionally accepted practices including Western norms that position men as family providers, non-emotive and aggressors within cathectic relations. *Subordinate *forms of masculinity typically embody non-masculine practices or effeminacy including domesticity, weakness and lack of authority. *Marginalized *masculinities are often linked to race, class and ethnicity and practices that are de-privileged because they do not conform to masculine ideals.

Cigarette smoking is often used to facilitate interpersonal interactions, and intimate couples are no exception. Critical gender perspectives have been useful for understanding smoking patterns among couples in a number of studies. Alignment with idealized masculinities has been shown to mediate a father's decision to continue smoking after the birth of a child [10-13]. A gender analysis of women's responses to men's smoking also revealed that new mothers aligned themselves with emphasized and ambivalent femininities and took up roles of regulating and defending fathers' smoking [14]. Although smoking and heavy drinking among men and women inhabiting similar social roles has been associated with higher masculinity scores [15], a longitudinal study of smoking behaviour, class and gender role identity reported more complex relationships between smoking and masculinity and femininity scales [16]. In general these studies point to the need for further understanding masculinity and femininity constructs and gender relations in relation to health behaviours, such as tobacco use.

### Parenting Trends and Family Dynamics

Parenting practices constitute specific enactments of gender relations. The change in social patterns and shifts toward employed mothers and involved fathers over the last several decades has been accompanied by changing expectations related to mothering, fathering and gender. The concept of the "new father" has emerged as a modern man who wants to be actively involved in direct childcare [17,18]; a distinctly different model than most men's own fathers [19,20] in operating outside the "distant, provider-disciplinarian to the more engaged and emotionally-expressive father" [[21], p.130]. Some authors position the new father as a mother's "helper" [22], whereas co-parenting ideally affords shared responsibility and leadership between the two parents [23]. Research on parenting demonstrates the potential importance of considering the influence of masculinities, femininities, and gender relations in the management of tobacco use in child-rearing families.

The interplay between fatherhood and masculinities, and a shift away from masculine ideals that may constrain men as stoic breadwinners has been described in interview-based research with fathers [24]. The men's experiences reflected the tensions and contradictions in juggling the multiple masculinities inherent in contemporary fathering; they felt the responsibility to be breadwinners but experienced it as an obligation that interfered with spending time with their children. These men engaged in childcare with enthusiasm, and maintained careers, but had little interest or responsibility for household work. These study findings suggest that working class men continue to be unreflexively complicit in reproducing hegemonic masculine ideals in the home.

Researchers examining couple relationships and the division of labour also point to the influence of gender ideology. A review of parenting among dual earning couples suggests there is evidence for increasing role balance in dual earner couples, and that couple attitudes or gender ideology are a major factor in maintaining this balance [25]. For example, in one study, fathers of infants under one year of age were more likely to be engaged in childcare if the mother's attitudes were feminist or non-traditional [26]. Fish, New, and Van Cleave [27] found that couples who report sharing childcare were more likely to perceive they had an egalitarian relationship and express satisfaction with the division of household tasks than traditional couples where women were the primary caregivers for the child. However, among shared couples, researchers found that women took more responsibility for tasks including food preparation, liaison with the school, and health-related tasks. This study exemplifies how alignment with gender ideology influences how couples perceive their relationships and division of labour. Men and women in shared couples overestimated the amount of the husband's contribution to household work because they perceived an egalitarian arrangement, and men and women in traditional couples minimized the amount of household work men were doing [27].

Egalitarian domestic arrangements are believed to produce better family communication and hypothesized to balance men's traditional socialization with qualities such as sensitivity, developed as a result of care giving [28]. Fathers who express high levels of nurturance and related qualities model an alternative to dominant ideals of masculinity, and similarly mothers who express autonomy and independence model an alternative to traditional femininities. Egalitarian parenting is also practiced by couples who ascribe to non-egalitarian gender ideologies, but for functional reasons, such as childcare costs for example, construct egalitarian domestic scenarios [28].

### Spousal dynamics, substance use and tobacco cessation

Intimate relationships strongly influence individual health behaviours, yet few studies approach smoking cessation research from the couple/dyad perspective to explore the bidirectional impact partners or spouses exert on each other. Women and men are more likely to quit smoking if their partner is a non-smoker, and those who smoke fewer cigarettes are also more likely to quit [29,30]. The challenges male smoking partners pose for pregnant women and new mothers who want to remain quit have been described [10,14], and researchers have underlined the necessity of including male partners in smoking cessation research [31].

Within heterosexual relationships, patterns of gender influence in terms of health behaviour differ markedly between men and women, and are dependent on gendered social relations. For example, wives' alcohol use is predictive of husbands' drinking levels after marriage, whereas prior to marriage, husbands' drinking is predictive of wives' consumption levels [32]. With marijuana, the pattern of gender influence is similar, except that wives also influence husbands' substance use during the transition to marriage, as well as during the early years after marriage [33]. This pattern of women's influence on men's health is also reported in smoking cessation research; men who perceive increased spousal influence to quit smoking report greater cigarette reductions; however, this pattern does not hold true for women [34]. These patterns are congruent with women's perceived role as primary family caregiver, a construct and practice anchored in feminine ideals about nurturing [4,14,35,36].

In conclusion, research has not generally separated healthcare tasks from childcare and household work in studies investigating the division of labour in families. Initial research indicates family healthcare tasks fall to the responsibility of women and more closely resemble household tasks in this regard [36]. Understanding tobacco use in intimate relational contexts, as it is influenced by gendered identities and power differences, remains an understudied component of everyday family experiences. The **objective of this study **was to examine how parenting young children and gender relations reflected in couple dynamics influence household tobacco use patterns and, specifically, women's tobacco reduction efforts.

## Methods

This study was part of a longitudinal, qualitative program of research investigating the micro-social context of tobacco use in families and employing a constructivist, gender relations perspective. Grounded theory methods [37] were used to systematically explore the underlying social processes involved in negotiating tobacco use within the context of parenting. The study took place in a large city in western Canada known for its smoke-free culture and tobacco control regulations in public spaces. The study protocol was approved by the University of British Columbia ethics review board.

### Sample

Study participants were recruited using local newspaper advertisements and from postnatal units of a large hospital. The couples who were invited to participate met the following inclusion criteria: a) they were living in a heterosexual relationship in the same household; b) the mother self-identified as a smoker who either quit or reduced smoking during her pregnancy; and c) the woman's pregnancy resulted in the birth of a healthy infant(s) at least one year prior to recruitment. The father's smoking status was not a factor that was considered in couple recruitment. We specifically recruited women who had quit or reduced during pregnancy because we were interested in how tobacco reduction efforts initiated in pregnancy were influenced by early experiences of parenting and couple dynamics. Twenty-eight female participants and 27 male partners provided written consent to participate in the study, and each received a $25 honorarium per interview to acknowledge their contribution to the research project. One father did not participate in the study; however, his demographic information was obtained from the mother's demographic survey and interview. For a description of the sample see Table 1.

**Table 1:** Characteristics of the Study Sample.

	Women (n = 28)	Partners (n = 28)
Age (years)		
20-29	8	5
30-39	20	20
40-49	-	3
Reported individual income		
< $10 000	5	1
$10 000 - $30 000	6	3
$30 000 - $50 000	6	6
$50 000 - $70 000	2	5
$70 000 - $90 000	-	1
> $90 000	-	1
Unknown	9	11
Ethnicity		
Anglo-Canadian	13	17
Eastern European	4	3
Asian/South Asian	8	4
Aboriginal/First Nations	2	2
Latino	1	-
Caribbean	-	1
South African	-	1
Parent smoking status (before pregnancy)		
Daily smoker	26	16
Occasional smoker (10 ≥ a week)	2	2
Non-smoker	-	7
Ex-smoker	-	3
Parent smoking patterns during pregnancy to early childhood		
Non-smoker	-	7
Ex-smoker		3
Quit and maintained quit:		
During pregnancy/postpartum	13	2
At year 1	-	1
At year 2	-	1
Reduced and maintained reduction	2	1
Quit/reduced and relapsed	13	2
Smoker (unchanged)	-	11

The 28 women participants had reduced or quit smoking during their pregnancy and their partners included 18 men who were smokers and 10 non-smokers or ex-smokers. Sixteen of the mothers maintained their quit/reduction status at the time of the last study interview, and twelve mothers had relapsed or continued their tobacco use at last contact. Among the smoking fathers, 5 of the 18 had not changed their smoking practices and were smoking at the time of the last interview, and 7 initiated a reduction in their level of smoking either during their partner's pregnancy or the early childhood period. Four fathers quit smoking; two during pregnancy, two immediately following the birth, and one at year 1 and another at year 2 after the birth of the baby. Two fathers who co-quit with mothers relapsed after birth, while their female partners maintained cessation.

### Data Collection

Individual semi-structured interviews were conducted by five trained research assistants (including one male interviewer) and were digitally recorded and transcribed. Individual interviews provided the opportunity for open disclosure of information on the part of participants, which may not have occurred in couple-based interviews [38]. Multiple interviewers allowed interviews to be conducted at the convenience of the participant; to match gender between interviewer and participant; and facilitated multiple perspectives to validate the direction of the analyses. Interviews were between 45-90 minutes long and took place at a location chosen by participants. Interviewers made field notes detailing general impressions of the interview and preliminary interpretations of the data.

Interviews were conducted at two time points related to the child's age: time point 1 (12-14 months-old) and/or time point 2 (24-42 months-old). Eleven dyads were interviewed at time 1, 14 dyads were interviewed at time 2, and 3 dyads were interviewed at times 1 and 2. Although we initially attempted to interview couples at time 1 and time 2, difficulties in follow-up data collection (1 year later) with young mobile couples made this challenging. Therefore, couples with young children (24-42 months of age) were also recruited and in addition to learning about their recent experiences with respect to smoking we collected retrospective data about earlier experiences. Data collected at point 1 highlighted mothers' and fathers' experiences and tobacco-related experiences in parenting during the first year following the birth of their child. During this period parents usually return to work and the couple must negotiate the division of household responsibilities and childcare. Interviews at point 2 captured retrospective data on first year parenting experiences, and further changes in parents' life experiences and tobacco use since their child reached one year-old. Participants were asked to discuss the organization of their daily lives as new parents. For example, we asked mothers and fathers, "What has changed in your household since your baby was born and now that she/he is # years old? How have you found the transition in terms of feeding the baby, work/employment, and childcare arrangements? How would you describe your responsibilities in the home as a father/mother over the past year? Participants were also asked to discuss their interactions with their partners and children, and whether these interactions undermined or promoted tobacco reduction. For example, they were asked how changes in family routines affected smoking decisions, the way issues related to smoking were dealt with as a couple, and whether these had changed since their child was born. Questions were also posed to explore changes in smoking restrictions in the home and car during pregnancy, the postpartum period and early childhood, and who was responsible for communicating the rules and negotiating smoke-free space for their child in other settings. Finally, women and men were asked how their partners influenced their efforts to reduce/quit smoking and keep their home smoke-free. The interviews were conducted over a period of 24 months. This provided time for data analysis following interviews and subsequent refinements of the interview questions to explore emerging themes and topics.

### Data Analysis

Interview transcripts and field notes were read by the authors and discussed within the investigative team to identify coding categories from which dyad (mother-father, mother-child and father-child interactions) summaries could be built [3]. The five research assistants who conducted the interviews were involved in data analysis; two of these individuals are co-authors (AC, MK). Open coding was used with initial interviews and dyad summaries to assist with organizing related passages and identifying underlying patterns and themes. Particular attention was paid to how parenting styles influenced the smoking practices of mothers and fathers. As additional interviews were analyzed open codes were refined and formed the basis of a coding framework. Emerging questions from the analysis were incorporated into subsequent interviews. NVIVO 8™ software was used to code all dyad summaries and facilitate data extraction. Because all of the women in our sample had reduced or stopped smoking for pregnancy, we were interested in how women's tobacco use was shaped by gender relations in the context of parenting young children. In the next stage of analysis we employed Howson's [9] model of gender relations as an analytic framework, guided by his conceptual categories of *emphasized*, *ambivalent *and *protest *femininities and *complicit, marginalized *and *subordinate *masculinities. Using this framework, we examined coded data to determine how constructed mothering and fathering practices were aligned and misaligned with gender ideals and influenced couples' tobacco use. Analytic categories were compared and contrasted to reveal patterns. Subsequent review of the analyses allowed members of the investigative team to collaboratively develop conceptual categories that represented the social processes investigated in this study.

## Results

Dyad smoking patterns varied in relation to parenting style. In particular, women's successes with maintaining tobacco reduction achieved during pregnancy through the postpartum period and into early childhood were influenced by parenting style and accompanying discourses of femininities and masculinities.

### Parenting styles

We identified two parenting styles across all the dyads, *traditional *or *shared*, based on how the dyad divided responsibilities for three domains: earned income, domestic work and childcare. See Table 2 for the details of parenting style classifications. The major distinction between the two categories revolved around the degree of the father's involvement at home, an approach common in the sociological literature on division of labour in households [27]. Others have reported that fathers may overestimate the amount of work they perform in the home [39] and when dyad accounts were not congruent, we relied on details of the mother's interview to make a classification.

**Table 2:** Parenting Style Classifications - Traditional and Shared.

*Traditional Parenting* Dyads that ascribe to traditional gender divisions of labour.		
	**Mother**	**Father**

**Income**	Mother has no responsibility for income generation, or her income and job provides a secondary economic contribution (e.g. works part-time or at lower wage), or she works fulltime and provides shared economic responsibility.	Father is usually the primary breadwinner, or he may have shared responsibility for income generation through employment or social assistance income.

**Chores**	Mother is primarily responsible for daily domestic chores, particularly indoor chores such as cooking and cleaning.	Father has little or no responsibility for daily domestic chores or cooking. He may perform outdoor chores such as garbage, yard work, renovations.

**Childcare**	Mother is primarily responsible for daily childcare routines. If the mother works, she is also responsible for managing most of childcare responsibilities.	Father has no regular responsibility for daily childcare routines. Secondary responsibilities are seen as 'helping the mother out' because the child is primarily her responsibility. The father may perform play activities with the child at times.

***Shared Parenting*** Dyads wherein the father shares a significant role in childcare responsibilities and/or domestic chores.		

	**Mother**	**Father**

**Income**	Mother may have primary or shared responsibility for income generation.	Father may have primary or shared responsibility for income generation.

**Chores**	Mother has primary or shared or secondary responsibility for daily domestic chores, such as cooking and cleaning.	Father has primary, shared, or secondary responsibility for domestic chores. For example, he may cook regular meals or be responsible for dishes.

**Childcare**	Mother has primary or secondary responsibility for daily childcare routines.	Father shares childcare responsibilities with mother or provides secondary responsibilities on a regular basis. For example, he may perform childcare duties on specified days of the week or hours of each day, while the mother works; or he may perform childcare duties on a regular basis while at home with the mother.

The practices of *shared *parenting dyads were viewed as challenging hegemonic masculinity, and were accompanied by *ambivalent *or *protest *femininities in the case of reluctant fathers; however, when members of a *shared *dyad embraced new fatherhood practices, we found less evidence of such gender relations. The practices of *traditional *parenting dyads formed by the pairing of *complicit *masculinities with *emphasized *femininities tended to sustain hegemonic masculinity. In some instances, we recognized that this gender pattern resulted as an artefact of patriarchal structures (i.e., opportunities for better paid jobs for men) and reflected mutual negotiation; however, gender relations comprised of *complicit *masculinities and *emphasized *femininities could become a site of tension over time.

Fifteen couples' parenting practices reflected the *traditional parenting style*, and most of the women in these dyads relapsed to smoking following pregnancy. Thirteen couples organized their lives in accord with a *shared parenting *style and were more likely to include women who maintained their tobacco reduction following pregnancy, and became dual non-smoking parents.

### Women who Sustained Cessation or Tobacco Reduction

**Figure 1:**
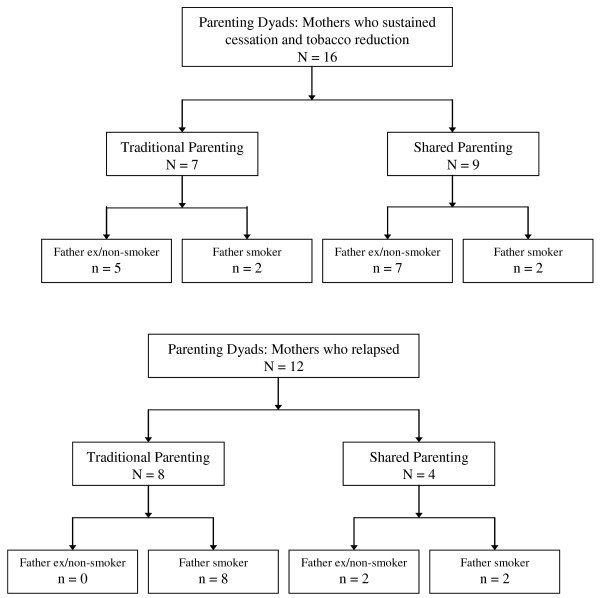
**Parenting style and smoking status**.

Among the 16 women who sustained their cessation or tobacco reduction up to two years following the birth of their infants, seven were involved in *traditional parenting *and nine in *shared parenting *(see Figure 1)

#### Traditional Parenting

Women involved in traditional parenting included five mothers who quit smoking during pregnancy and remained smoke free for up to two years postpartum. Two women maintained significant reductions in their smoking during the same time period (a reduction to 1 cigarette per day [CPD] from half a pack and 4-6 cig/day from 10-12 CPD pre-pregnancy, respectively). The smoking status of their partners/husbands included three fathers who had never smoked, one father who quit one week before the birth of his child, and another who quit when his child was one year old.

In their narratives these women aligned with *emphasised femininities; they *accepted full responsibility for childcare and the domestic work. Their male partners aligned with hegemonic masculinity; they all worked outside the home and had minimal involvement in domestic activities. Although smoking was a non-issue for most of the couples in this group because of their smoke-free status, all who quit or reduced smoking for pregnancy or parenthood remained vulnerable to relapse. Participants described experiences of cravings, worry about occasional slips, difficulties in remaining smoke-free, and concern about returning to activities that involved regular smoking in the past. For women, vulnerability to relapse appeared to be related to the stress of managing childcare along with the demands related to returning to paid work. For men, vulnerability to relapse was associated with being "out with the boys." As such, there was an undercurrent in the narratives of the need to be vigilant. Women in particular felt pressure to maintain their tobacco reduction. As one woman stated, smoking mothers are "more frowned upon" than fathers, because women are the primary caregivers. And, as the partner of a mother who spent long hours alone at home with her child said of the woman's tobacco reduction, her smoking is "self-regulated" by childcare responsibilities, and she "only smoked" outdoors. Smoking restrictions at home and in the car supported household tobacco reduction efforts, and were deemed as relatively easy to implement -- most couples no longer smoked indoors.

Although mothers' ability to remain quit or maintain reductions in this traditional parenting subgroup supports literature documenting how women's partners' smoking status influences their ability to remain quit [10], gender influences reinforced by a traditional parenting style also appeared to play a key role. Strongly related to *emphasized *femininities, women in this group subscribed to the conventional notion of the "good mother" [39]. Strong alignment with this gender discourse precluded a return to daily smoking, and resulted in women leading the couple towards tobacco reduction.

Hegemonic masculinity also characterized gender relations among *traditional parenting *dyads albeit in less visible ways. Two of the women, both long-term daily smokers, had successfully maintained their quit status at the time of interview 2, but were partnered with men who smoked occasionally. These women accepted that their breadwinner partners smoked on infrequent, social occasions, always with their permission. The partner's occasional smoking was situated as reward-based incentives for meeting their family obligations.

#### Shared Parenting

Women involved in shared parenting included 8 mothers who quit for their pregnancy and remained quit at two years postpartum, as well as one mother who reduced during her pregnancy and maintained a reduction. The mothers in this group were clear about their resolve related to smoking, as one mother stated: "I just really believe that cigarette smoke and babies don't mix." We speculated as to whether *shared parenting *practices may have more readily facilitated mothers' and fathers' successful transition to smoke-free homes and families; however, a closer investigation of the dyad summary data revealed how alignment with masculinities and femininities added a layer of complexity to this transition.

A dual smoking couple's successful transition to becoming dual smoke-free parents after smoking together for 15 years showed how contemporary parenting discourses mediated the father's alignment with masculine ideals. Both parents in this dyad described highly egalitarian parenting practices; after the birth, the couple rearranged their work lives so that one of them was always at home with the child, and this resulted in the father running his carpentry business in the evenings so that he could care for the child during the day when the mother worked. The first time mother was steadfast to staying smoke-free, "I told myself for years, I will not be a smoking mother of a young child." She maintained her cessation alone for almost two years during which she continually encouraged her partner to quit smoking. Only when she threatened to return to smoking if he did not become a smoke-free parent did the father join her by quitting smoking. The father emphasized how parenthood and supporting his partner's quit attempt was the sole reason for giving up the pleasure of smoking: "Let's say if I would live alone, probably I would smoke. Not probably, I'm pretty sure." He also stated, "I felt kind of responsible, you know, after two years it would be too sad to, to not give her at least a chance to stay smoke free" The man's comments suggest that his acceptance of new fatherhood values mediated and conflicted with his alignment to *complicit *masculinities. As well, we noted that the mother led the quitting efforts within the context of a *shared *parenting dynamic, pointing to gender interactions characterized by *ambivalent *(shifting to *protest) *femininities (her ultimatum) that strategically relied on social acceptance of the responsibilities in the new father role to modify *complicit *masculinity, as presented by her husband.

Similarly, another mother from a shared parenting, non-smoking dyad, who prior to her pregnancy had a 20-year smoking history and numerous unsuccessful cessation attempts, quit smoking during her pregnancy, and was followed in the quit attempt several months later by the father. At interview time 2 the couple reported they had remained smoke-free together. The mother described the couple's parenting practices saying, "We're pretty much equal in everything, in all our decisions and stuff."

Although both these couples embraced a lifestyle focussed around shared parenting that entailed significant father involvement at home, the mothers in most instances led the decision to become a smoke-free family. This suggests that shared and egalitarian parenting practices may position women to exert more influence around family decision making [40]. However, it does not negate the potential for male partners to take an active role in ensuring women remain smoke free. In one dyad, a mother (and professional lawyer) eliminated the conflict over her smoking by quitting during pregnancy and remaining quit. Although she was adamant that she "always knew" she wouldn't smoke as a mother, her anti-smoking husband was cognizant of the possibilities of relapse. He stated that if anyone encouraged his partner to smoke again he would, "step in pretty quickly and tell them to beat it." In this instance, we can read the voice of the new father drawing on hegemonic masculinity to defend and maintain the good mother. During this man's interview, we queried what would happen should his partner relapse and he stated that he viewed such a decision as grounds for divorce. We encountered this same perspective from non-smoking fathers in our previous research on women's smoking and pregnancy [10].

In two dyads in this *shared parenting *group, the fathers remained smokers over the duration of the study. It is possible that use of tobacco constitutes a familiar mechanism for men vested in maintaining traditional masculine identities as they transition into fatherhood. Nonetheless, the dyads illustrated the friction and discomfort that developed when smoking practices reflective of hegemonic masculinity lingered in a shared parenting dynamic. For example, in one of dyads the mother successfully quit during the pregnancy and was the only participant who maintained her cessation with a spouse who was a daily smoker. Interestingly, this woman was also the main breadwinner for her family and returned to work before her maternity benefits expired demonstrating a break with conventional motherhood and *emphasized *femininities. She attempted to obtain an agreement from her partner that he would stay home and perform fulltime childcare during the first year, however, he retracted this agreement stating he preferred to work in construction part-time. She was clear about her position on smoking, "I think that if you have kids you should make the decision to stop smoking for the good of your family and just stop." Accordingly, she continued to lobby and demand her husband to quit (reflective of *protest *femininities). She eventually conceded, however, by recognizing his reduction from a pack to half a pack a day and reluctantly accepted that he would continue to smoke, perhaps as a strategy to reduce the tobacco-related conflict that was evident in their marriage. We conjectured that continued smoking in the context of *shared parenting *produced conflict because both partners had become more vested in daily childcare and domestic routines, thus making tobacco use more visible because it was no longer contained in separate gendered spheres.

The other mother in a *shared parenting *dyad with a father who smoked reduced rather than quit for her pregnancy. The couple had reduced together during the pregnancy and remained a dyad of light smokers at time 2, however, the father always smoked slightly less than the mother (1-2 CPD). Although the mother had reduced from half a pack to 2-3 cigarettes a day during the pregnancy, she increased her smoking levels to 4 CPD during the first year postpartum. At that same time her maternity benefits came to an end and each parent was working part-time and sharing childcare responsibilities equally. This dyad, who also smoked marijuana together, was an unusual classification in our sample and we viewed the mothering of this 22-year-old mother as aligned with *ambivalent*, if not *protest *femininities. She stated that the "big urge to quit" associated with pregnancy had past, that tobacco was her "security blanket right now," and that smoking had a place in her family. Her partner hoped that their childcare responsibilities would foster a "natural reduction" over time. Smoking for this young woman seemed to represent the freedom and rebellion of youth without the responsibilities of motherhood and represented a means to protest social norms for women and mothers. From a gender relations perspective, this father may be either seen as promoting tobacco reduction in a conciliatory fashion of a *new father*, or as following the mother's lead to maintain his own continued smoking.

In summary, shared parenting practices were linked with a commitment to tobacco cessation with less acceptance of men's smoking, and alignment with gender discourses that challenged hegemonic masculinity.

### Women who Relapsed to Smoking

Among the 12 mothers who relapsed to former or increased smoking levels, 8 practiced *traditional parenting*, and 4 practiced *shared parenting*.

#### Traditional Parenting

Eight mothers who relapsed to former smoking levels after their pregnancy lived in traditional parenting families with fathers who smoked and expressed resistance to quitting. Mothers in these dyads usually smoked less than the father, and in their role as primary caregivers monitored tobacco use and smoke-free house rules. Although they tried not to "nag" their partner about reducing or quitting, women led efforts to create smoke-free environments for their children. A 30-year-old father working in retail, self-described as "reducing," was married to a stay-at-home mom who smoked "casually." The mother had quit for her pregnancy, but relapsed two weeks after the birth by smoking with her husband in the evening. She had expressed how much she wanted them to co-quit, but he indicated how quitting ceased to be an option:

Because my wife smokes less compared to myself it's more on me. She makes it quite clear she's unhappy with me smoking as much as I do...And I've made it quite clear to myself that I need to cut down... Especially since [baby] was born there's times when she's 'okay you're smoking too much' and at that point I stop. It's not worth having the argument. Or I just wait until she goes to bed. It's one or the other. But we both set out standards not to smoke around the kids and as long as we're keeping to that...that's what matters most.

We observed that *traditional parenting *dyads put mothers at higher risk for relapse. Women in these dyads aligned with *emphasized *femininities, enabling them to act as a supportive and harmonious partner, and refrain from demanding their partners quit tobacco use. The women had either reduced or quit smoking for their pregnancy, but did not wish to disrupt relationship dynamics by insisting on cessation. Men in these dyads aligned with hegemonic masculinity, ignoring the health risks of tobacco use, and did not find fatherhood strong enough motivation to quit.

Although parenting practices were separate, gendered activities, smoking was often a joint activity that couples enjoyed together, and men and women reiterated how co-quitting would be essential for cessation. In addition, these traditional dyads voiced agreement with the notion that secondhand smoke is a health threat, thereby attempting to be viewed as "good parents" without undergoing tobacco cessation. These shared understandings facilitated the couple's continued smoking and minimized pressure to change.

#### Shared Parenting

Four women in *shared parenting *dyads relapsed to former or increased levels of smoking. In two of these dyads, the mothers' continued smoking was the source of couple conflict, and both fathers used coercion to persuade their wives to quit, threatening plans for having a second child were conditional on the complete elimination of smoking. One of these mothers, a 30-year-old receptionist, said "it's a bitter topic", and stated that the couple's interactions related to tobacco became worse after the pregnancy, because her husband had exerted more pressure on her to quit and remain quit. Both fathers also purchased the nicotine patch for their wives and attempted to insist it was worn. In both dyads, the couples experienced significant tension and conflict in their marriage over the woman's continued use of tobacco in conjunction with raising young children. This conflict appeared to be more marked than conflict observed among traditional parenting dyads whose daily life often consisted of paid work-home gendered divisions of labour. Both men in these dyads were highly engaged in shared parenting, and the notion of smoking and parenthood posed an incompatible reality for them. Their alignment with the *new father *prompted a desire to be matched by a partner defined as a *good mother*, and therefore, smoking posed a challenge to these gender relation ideals.

Rather than positioning the man as the culprit, we interpret the gender dynamics in more nuanced terms, as a joint struggle at cross purposes - he demanding a concession (quit) from her in return for being a *new father*, she protesting the hegemony of the *good mother*. These dyads were the most conflictual in the sample, a complex intertwining of competing gender, parenting, and smoking agendas.

Two dual-smoking dyads fit the *shared parenting *criteria as a result of economic or childcare circumstances, and an expressed desire to return to their former *traditional *family arrangements. For example, one father was at home on disability due to a work accident that occurred during the pregnancy, forcing his partner to work outside the home for the first time in 10 years. Both the mother and father attributed the perceived stress of this new arrangement to be the reason for their increased smoking levels since the birth of their child. During the pregnancy the mother had reduced to half a pack a day, and at the time 2 interview she was working fulltime and smoking a pack a day. Although no longer the primary care giver for her children, the mother's alignment with traditional motherhood was apparent:

I have to have to pull myself away from that and go, "Okay I've got to leave him in daddy's hands now, go off and be independent working mom." [And] that was really hard for me because I was able to stay at home with all the other ones through their first steps.

The father had been a non-smoker at times in the past, but began smoking around a pack a day when he assumed daily responsibilities for the couple's newborn and three older children. The father was adamant that "we should quit," a sentiment not reflected by the mother who insisted she could not quit because of the "stress of being a working mom" and trying to juggle her "newfound career and old career of being a mom." This couple exemplifies the way smoking patterns are influenced by parenting styles and how these may not be in accord with gender-based values.

## Discussion

This study demonstrates that attention to gender relations provides a very useful way of understanding behaviour such as tobacco use. The findings suggest that a couple's parenting style reflect performances of femininities and masculinities and were associated with particular smoking patterns. *Traditional parenting *reinforced by women's alignment with emphasized femininities and men's alignment with hegemonic masculinities placed women with smoking partners at risk for relapse. Women's actions to be supportive partners facilitated couples' continued smoking and placed those who remained smoke free vulnerable to relapse. In *shared parenting *dyads, egalitarian practices tended to support successful transitions to smoke-free homes. Women's ability to exert more influence around family decision making related to smoking, and alignment with gender discourses associated with new fatherhood that challenged hegemonic masculinity were influential. Although women frequently led efforts to become smoke-free families, fathers involved in shared parenting also took an active role in supporting women's cessation and were more likely to reduce or stop their own smoking. It appears that shared parenting styles mediated by shifts in performances of femininities and masculinities that accompany such practices support commitments to tobacco reduction and cessation.

The interconnectedness of parenting styles, gender relations, and the tobacco use patterns support efforts to examine the influence of household dynamics and the limitations of models of smoking cessation that focus on individual behaviour change. In addition to the influence of dominant discourses related to gender, factors such as employment, and access to resources played an important role in shaping the gendered dynamics relating to parenting and tobacco use. Although the full range of interaction and parenting styles may not have been captured by the study sample, in-depth interviews with both members of the dyad and their accounts of experiences over time provided a rich source of data for this study. Insights related to the importance of gender relations add to a growing field of research documenting the influence of gender on health and reinforce the need to consider gender influences in tobacco control research.

The findings indicated that motherhood and smoking were more problematic than fatherhood and smoking, and that gender relations and parenting reinforced these differences. This was not surprising, for two reasons. First, there has been a well established history of attention to the issue of smoking during pregnancy, spanning forty years [41-43]. This attention has precluded attention to broader issues concerning women's health and women's smoking, by focusing on the responsibility of women to ensure fetal and infant health [42,43]. Second, there appears to be considerable stigma attached to maternal smoking, particularly during pregnancy [44] which may become more complex as smoking continues to be denormalized. Comparatively little has been said or done to intervene with fathers' smoking, or expectant fathers' smoking, and even less about these gender differences in perception and social attitudes and how they are absorbed and acted upon by women and men in couples.

The finding it was women who generally led efforts for the couple to quit or reduce smoking is supported by other research with respect to alcohol where women led health-related behaviours after marriage [32]. Although there has been attention to the influences that women have on men's health [35,45], a more nuanced understanding about how men inform or govern the health practices of women (and children) is needed. While this likely relates to dominant discourses espousing the incompatibility of masculinity and self-care, our findings suggest that men can and do operate in subtle (and occasionally not so subtle) ways to direct and/or co-construct parenting and the health practices of women partners. As described in our findings, the influence of men and dominant masculine ideals about gender relations permeate parenting practices in both traditional and shared configurations. Present here are dominant ideals about gender relations in which smoking and parenting connect to masculinities and femininities to collaborate, compete or contest power differentials.

It is tempting to argue that fathers from our shared parenting dyads were resisting traditional discourses of fatherhood, and indeed, fatherhood offered some men in this study an opportunity to expand masculine identities; their uptake of shared parenting responsibilities points to the ongoing social change related to masculinities. Family researchers have argued that whether men purposely align with less traditional masculinities or not, "the reality is, however, when fathers are at home caring for young children they become nurturers themselves, however much that contradicts their gender beliefs" [[39], p. 26]. This argument complements research that has demonstrated how men's greater social-psychological commitment to fathering results in positive changes to men's well-being and relationship with the mother [46]. In terms of tobacco use, this is certainly the case. The more men in this study became involved fathers and caregivers, the more likely they were to reduce or quit smoking.

Other research focussed on social interactions between new parents has showed that fathers' increased involvement with their child is also shaped by the mother. In addition, agreements over the division of labour related to housework (as opposed to child care) provokes the most conflict for couples, especially in households where mothers refuse to assume this conventional responsibility [47]. In addition, the evidence that family healthcare decisions bear most similarity to divisions of labour related to household work [36], and that housework continues to be entrenched by gender ideology as "women's work" [47,48] is in keeping with our finding that changes in household tobacco use and practices, like other health-care responsibilities, were led by mothers. This finding adds a layer of complexity in demonstrating how men and women influence each other, and how gender relations mediate health-related decisions.

Our findings indicate that power/control dynamics expressed through parenting styles continue to influence tobacco reduction beyond pregnancy and early postpartum period. This analysis augments our earlier observations [49] and indicates that such dynamics can be subtle in nature, ongoing, and not necessarily situational. In general across all the non-smoking dyads where the mother, father, or both reduced or stopped smoking, we observed the subtext of potential conflict in the event either mother or father relapsed. There was also evidence of tension and conflict in shared parenting couples where women continued to smoke and partners wanted them to quit. These findings were somewhat surprising because we had assumed after the pregnancy, couples might experience less conflict over smoking; however, perhaps denormalization, anti-smoking norms and stigma faced by parents who smoke is such that tobacco-related conflict is potentially an ongoing issue.

### Study Implications

The study findings provide some directions for interventions to support smoke-free families. Interventions that support shared parenting and engagement in fathering may be a useful complement to efforts to support tobacco reduction efforts in child rearing families. Smoke-free family initiatives also need to include support for men's tobacco reduction to assist women in their own tobacco reduction and their efforts to establish smoke-free homes. The potential for conflict/tension related to tobacco use, especially in light of vulnerabilities to relapse long after the immediate postpartum period, suggests that delinked interventions (i.e., separate interventions for men and women) to support tobacco reduction need to extend well beyond pregnancy/postpartum. We have argued elsewhere for delinked interventions to support tobacco reduction in couple dyads that include information about couple dynamics related to tobacco and foster conciliatory efforts to achieve a smoke free family status [10]. The importance of using a gender sensitive approach is also reinforced by our findings. For example, for many men cooperating with female partners around establishing practices to support tobacco reduction in specific locales, such as parenting and domestic spheres, called for some masculine deviance from assuming the 'power' position. Thus, supporting men's ability to reformulate hegemonic masculinity to foster collective shared parental power whilst avoiding the marginalized or subordinate masculine roles and identities that can accompany that shift may be helpful in engaging them in tobacco reduction.

## Conclusion

Understandings of how tobacco use is integrated in spousal and parental roles should be incorporated into tobacco reduction approaches. Decisions about tobacco use are made within relationships and social contexts that vary based on each individual's relationship to tobacco, divisions of domestic labour and childcare, and other activities that impact tobacco use. Therefore, solutions that propose communicating more strongly the biomedical health risks associated with smoking may not be effective, whereas, interventions that take into account gender relations may lead to better public health messaging and efforts to support tobacco reduction in families.

## Competing interests

The authors declare that they have no competing interests.

## Authors' contributions

JLB conceived and designed the study; awarded funding to carry out the study; guided the data analysis, and writing of the manuscript. MTK carried out data collection, contributed to data analysis, and writing of the manuscript. JLO contributed to the design of the study, the data analysis and writing of the manuscript. JLJ contributed to the data analysis and writing of the manuscript. LG contributed to the data analysis and writing of the manuscript. AC carried out data collection, contributed to data analysis, and writing of the manuscript.

## Pre-publication history

The pre-publication history for this paper can be accessed here:

http://www.biomedcentral.com/1471-2458/10/239/prepub
